# Supplementary material for: Asymmetric transformation of achiral gold nanoclusters with negative nonlinear dependence between chiroptical activity and enantiomeric excess
Source: Nat Commun. 2023 Jun 22;14:3730. doi: 10.1038/s41467-023-39462-w (PMC10287656; doi:10.1038/s41467-023-39462-w)
Supplement: Supplementary file 1 — Supplementary Information File [file 41467_2023_39462_MOESM1_ESM.pdf]

Supplementary Information

Asymmetric transformation of achiral gold nanoclusters with negative nonlinear dependence between chiroptical activity and enantiomeric excess

Chang Liu, Yan Zhao, Tai-Song Zhang, Cheng-Bo Tao, Wenwen Fei, Sheng Zhang and Man-Bo Li*

Institutes of Physical Science and Information Technology, Key Laboratory of Structure and Functional Regulation of Hybrid Materials of Ministry of Education, Anhui University, Hefei 230601 (P. R. China).

Chang Liu and Yan Zhao contributed equally

E-mail: mbli@ahu.edu.cn

Table of Contents

Supplementary Methods	S1-S4
Supplementary Tables	S5-S7
Supplementary Figures	S8-S20
Supplementary References	S21

Supplementary Methods

Reactions of Au_{23} with phosphines

(1) Synthesis of $[Au_{23}(SC_6H_{11})_{16}]^{-}TOA^{+}$

$HAuCl_4 \cdot 3H_2O$ (0.3 mmol, 118 mg) and tetraoctylammonium bromide (TOAB, 0.35 mmol, 190 mg) were dissolved in methanol (15 mL) in a 100 mL round-bottom flask. After vigorously stirring for 15 min, the solution color changed from yellow to dark reddish orange. Then, cyclohexanethiol (1.6 mmol, 196 μL) was added to the mixture at room temperature. The reddish orange solution turned yellowish immediately, indicating the conversion of Au(III) to Au(I) complexes. After 15 min, $NaBH_4$ (3 mmol, 114 mg dissolved in 6 mL of cold Nanopure water) was rapidly added to the solution under vigorous stirring. The solution turned black immediately, indicating the formation of Au clusters.[1] The reaction mixture was allowed to stir overnight and finally gave rise to Au_{23} (20% yield, Au atom basis), which was further purified by recrystalization.

(2) Synthesis of phosphoramidites

*Preparation of (**S**)-**L₁***

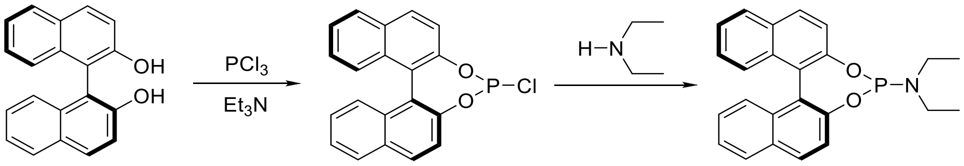

Triethylamine (5.0 eq., 25 mmol, 3.5 mL) was added dropwise to a stirred ice-cooled solution of PCl_3 (5 mmol, 436 μL) in dichloromethane (DCM, 35 mL). The ice bath was removed and the solution was warm to room temperature before diethylamine (5 mmol, 517 μL) was added. After 5 h, (***S***)-binaphthol (5 mmol, 1.43 g) was added to the suspension and the resulted mixture was left to stir for an additional 18 h. The purification of the reaction was achieved via column chromatography on silica gel (eluent: Pentane/EtOAc = 60/1) to give (***S***)-$\mathbf{L_1}$ (1.30 g, 3.4 mmol, 68%), colorless oil. ^{1}H NMR (400 MHz, $CDCl_3$) δ 7.98-7.90 (m, 4H), 7.52 (d, J = 8.8 Hz, 1H), 7.44-7.35 (m, 5H), 7.30-7.23 (m, 2H), 3.12-3.01 (m, 2H), 2.92-2.80 (m, 2H), 1.06 (t, J = 6.8 Hz, 6H). The ^{1}H NMR data is consistent with that in the reported literature.[2]

The general method from above was used for the preparation of (***Rac***)-$\mathbf{L_1}$, (***R***)-$\mathbf{L_1}$ and $\mathbf{L_{2\text{-}5}}$ with racemic, R and S configurations.

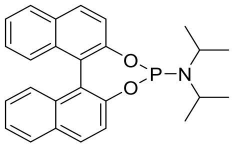

$\mathbf{L_2}$ (1.32 g, 3.18 mmol, 64%), colorless oil. ^{1}H NMR (400 MHz, $CDCl_3$) δ 7.92-7.89 (m, 4H), 7.52 (d, *J* = 8.8 Hz, 1H), 7.46-7.38 (m, 4H), 7.33-7.21 (m, 3H), 3.44-3.34 (m, 2H), 1.23 (d, *J* = 6.8 Hz, 6H), 1.19 (d, *J* = 6.8 Hz, 6H). The ^{1}H NMR data is consistent with that in the reported literature.[3]

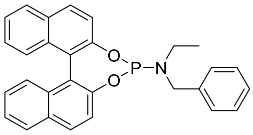

$\mathbf{L_3}$ (1.30 g, 2.90 mmol, 58%), colorless oil. ^{1}H NMR (400 MHz, $CDCl_3$) δ 7.98 (d, *J* = 8.8 Hz, 1H), 7.92 (d, *J* = 8.0 Hz, 1H), 7.88-7.85 (m, 2H), 7.57 (d, *J* = 8.8 Hz, 1H), 7.43-7.31 (m, 9H), 7.26-7.22 (m, 3H), 4.28-4.22 (m, 1H), 3.82-3.76 (m, 1H), 3.04-2.93 (m, 1H), 2.67-2.65 (m, 1H), 1.06 (t, *J* = 6.8 Hz, 3H). The ^{1}H NMR data is consistent with that in the reported literature.[4]

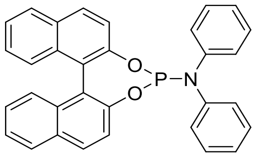

$\mathbf{L_4}$ (1.18 g, 2.44 mmol, 49%), colorless oil. ^{1}H NMR (400 MHz, $CDCl_3$) δ 7.96 (d, *J* = 8.8 Hz, 1H), 7.90 (d, *J* = 8.4 Hz, 1H), 7.75 (d, *J* = 8.4 Hz, 1H), 7.53-7.47 (m, 2H), 7.42-7.33 (m, 2H), 7.26-7.17 (m, 4H), 7.08-7.06 (m, 4H), 7.04-7.00 (m, 4H), 6.97-6.90 (m, 3H). The ^{1}H NMR data is consistent with that in the reported literature.[5]

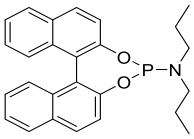

$\mathbf{L_5}$ (1.35 g, 3.25 mmol, 65%), colorless oil. ^{1}H NMR (400 MHz, $CDCl_3$) δ 7.96 (d, *J* = 8.8 Hz, 1H), 7.91 (t, *J* = 6.8 Hz, 3H), 7.51 (d, *J* = 8.8 Hz, 1H), 7.43-7.38 (m, 4H),

7.35 (d, J = 8.8 Hz, 1H), 7.29-7.22 (m, 2H), 2.95-2.85 (m, 2H), 2.82-2.72 (m, 2H), 1.54-1.44 (m, 4H), 0.78 (t, J = 7.2 Hz, 6H). The ^{1}H NMR data is consistent with that in the reported literature.[6]

(3) The reactions of different phosphine ligands with Au_{23}

Reaction of Au_{23} with butane-2,3-diybis(diphenylphosphine)

3.0 equivalents of butane-2,3-diybis(diphenylphosphine) (2.80 mg) were added into the DCM (1.5 mL) solution of Au_{23} (15.0 mg). The reaction was monitored by UV-vis. After 4 h, the mixture was purified by prepared thin layer chromatography (PTLC) with DCM/MeOH = 20/1. As shown in Supplementary Figure 1, at least three components were observed based on the PTLC. Their UV-vis spectra were also shown in Supplementary Figure 1a. A lower equivalent of the phosphine ligand or reaction temperature did not improve the selectivity either. These results indicated that butane-2,3-diybis(diphenylphosphine) with two chiral sp^3 carbons was reactive to Au_{23}, but showed poor selectivity and resulted in wide product distribution.

Reaction of Au_{23} with BINAP

5.0 equivalents of BINAP (6.83 mg) were added into the DCM (1.5 mL) solution of Au_{23} (15.0 mg). The reaction was monitored by UV-vis, and it was found that the UV-vis spectra kept unchanged even after four days. A higher equivalent of BINAP did not improve the reactivity either. The mixture was purified by PTLC with Pentane/DCM = 2/1 and Au_{23} was found to be recovered based on the UV-vis spectrum (Supplementary Figure 1b). This result indicated that axially chiral BINAP with sterically hindered phosphine sites displayed low activity to Au_{23}, and led to the recovery of the gold nanocluster.

Reaction of Au_{23} with phosphoric acid

3.0 equivalents of phosphoric acid (2.29 mg) were added into the DCM (1.5 mL) solution of Au_{23} (15.0 mg). The reaction was monitored by UV-vis, and it was found that the mixture turned to colorless quickly, demonstrating the decomposition of Au_{23} nanocluster. A lower equivalent of the phosphoric acid did not give better results. This

result indicated that phosphoric acid triggered the decomposition of Au_{23}, probably due to its strong acidity.

Reaction of Au_{23} with phosphoramidites ($\boldsymbol{L_1}$-$\boldsymbol{L_5}$)

5.0 equivalents of $\mathbf{L_1}$ (4.24 mg) were added into 15.0 mg of Au_{23}, which were dissolved in 1.5 mL of DCM. After the completion of the reaction in 4 h, the mixture was concentrated, and washed with MeOH for 2-3 times to give the crude $\mathbf{LC_1}$ (precipitate). Recrystallization of the crude product in the system of DCM/n-hexane gave red hexagon crystals (Supplementary Figure 2b), which were suitable for UV-vis (Supplementary Figure 2a), ESI-MS, SCXRD and the other characterizations. Notably, purification of the reaction was also achieved via PTLC or column chromatography on silica gel (eluent: DCM/MeOH = 20/1), giving the pure $\mathbf{LC_1}$ (10.0 mg, yield: 65%, based on Au atom). PTLC showed that three components were included in the products (Supplementary Figure 2c): component A (soluble complexes), component B ($\mathbf{LC_1}$) and component C (insoluble nanoparticles).

The procedure from above was also applied for the preparation of $\mathbf{LC_2}$-$\mathbf{LC_5}$ by using $\mathbf{L_2}$-$\mathbf{L_5}$. The reactions were isolated by column chromatography on silica gel (eluent: DCM/MeOH = 20/1) to give the yields of three components of products (Supplementary Table 2).

Supplementary Tables

Supplementary Table 1. Reaction results of different chiral phosphine ligands with Au_{23}

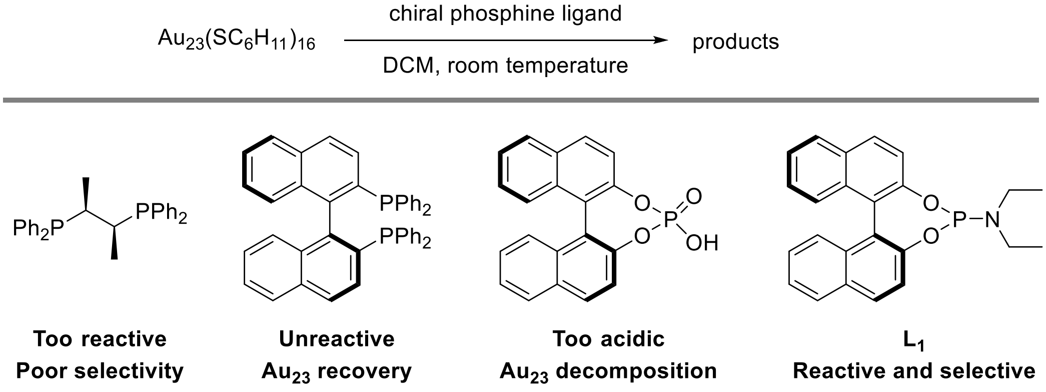

Supplementary Table 2. Reaction results of $\mathbf{L_1}$-$\mathbf{L_5}$ with Au_{23}

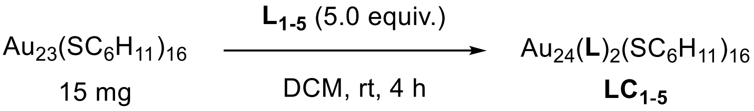

$L_{1\text{-}5}$	Yields of products		
	Component A (complexes)	Component B ($LC_{1\text{-}5}$)	Component C (nanoparticles)
L_1	3.0 mg	10.0 mg, 65%	1.3 mg
L_2	3.8 mg	9.20 mg, 59%	1.5 mg
L_3	3.3 mg	10.2 mg, 65%	1.3 mg
L_4	3.0 mg	9.00 mg, 57%	1.5 mg
L_5	3.5 mg	10.0 mg, 64%	1.4 mg

Supplementary Table 3. Amount of (***R***)-$\mathbf{L_1}$ and (***S***)-$\mathbf{L_1}$ used for the preparation of $\mathbf{L_1}$ with different ee

ee/% / L_1 (mg)	-100	-80	-60	-40	-20	0	20	40	60	80	100
(*R*)-L_1	0	1	2	3	4	5	6	7	8	9	10
(*S*)-L_1	10	9	8	7	6	5	4	3	2	1	0

Supplementary Table 4. Crystal data and structure refinement for **LC1**

	**(*R*)-LC1**	**(*S*)-LC1**
CCDC Number	2216153	2216156
Empirical formula	$C_{144}H_{220}Au_{24}N_2O_4P_2S_{16}$	$C_{144}H_{220}Au_{24}N_2O_4P_2S_{16}$
Formula weight	7345.30	7345.30
Temperature (K)	296.15	296.15
Crystal system	monoclinic	monoclinic
Space group	$P2_1$	$P2_1$
A (Å)	26.7353(18)	26.555(2)
B (Å)	33.348(2)	33.331(3)
C (Å)	43.034(3)	43.127(3)
Α (°)	90	90
Β (°)	108.051(2)	107.875(4)
Γ (°)	90	90
Volume ($Å^3$)	36479(4)	36329(5)
Z	6	6
Pcalcg (cm^3)	2.006	2.014
Μ (mm^{-1})	14.594	19.355
F(000)	19872.0	19872.0
Crystal size (mm^3)	0.1 × 0.1 × 0.08	0.09 × 0.09 × 0.1
Radiation	MoKα (λ = 0.71073)	GaKα (λ = 1.34139)
2Θ range for data collection (°)	3.43 to 55.136	2.97 to 102.498
Index ranges	-19 ≤ h ≤ 34, -41 ≤ k ≤ 43, -54 ≤ l ≤ 55	-30 ≤ h ≤ 24, -38 ≤ k ≤ 33, -45 ≤ l ≤ 50
Reflections collected	275282	194226
Independent reflections	157258 [R_{int} = 0.0843, R_{sigma} = 0.1358]	105347 [R_{int} = 0.1644, R_{sigma} = 0.1691]

Data/restraints/parameters	157258/17193/4910	105347/28014/5028
Goodness-of-fit on F^2	1.019	1.140
Final R indexes [I>=2σ (I)]	R_1 = 0.0841, wR_2 = 0.2103	R_1 = 0.0891, wR_2 = 0.2161
Final R indexes [all data]	R_1 = 0.1817, wR_2 = 0.2659	R_1 = 0.1794, wR_2 = 0.2494
Largest diff. peak/hole (e Å^{-3})	2.86/-2.00	3.12/-2.53
Flack parameter	0.224(9)	0.319(17)

Explanation for Alerts

(*R*)-LC$_1$

Alert level B

PLAT342_ALERT_3_B Low Bond Precision on C-C Bonds .... 0.03214 Ang.

Author Response: Large Uiso presented on the alkyl side chains, solvent molecules, and counteranions showed the potentiality of disorder, resulting in this alert.

(*S*)-LC$_1$

Alert level B

PLAT241_ALERT_2_B High ’MainMol’ Ueq as Compared to Neighbors of S1_16 Check
PLAT241_ALERT_2_B High ’MainMol’ Ueq as Compared to Neighbors of S1_19 Check
PLAT241_ALERT_2_B High ’MainMol’ Ueq as Compared to Neighbors of S1_54 Check
PLAT242_ALERT_2_B Low ’MainMol’ Ueq as Compared to Neighbors of Au18_2 Check
PLAT242_ALERT_2_B Low ’MainMol’ Ueq as Compared to Neighbors of Au22_2 Check
PLAT242_ALERT_2_B Low ’MainMol’ Ueq as Compared to Neighbors of Au23_2 Check
PLAT242_ALERT_2_B Low ’MainMol’ Ueq as Compared to Neighbors of Au21_1 Check
PLAT242_ALERT_2_B Low ’MainMol’ Ueq as Compared to Neighbors of Au22_1 Check

Author Response: These alerts are due to the disorder of the corresponding atom, and the temperature factor of an atom is higher or lower than that of nearby atoms.

Supplementary Figures

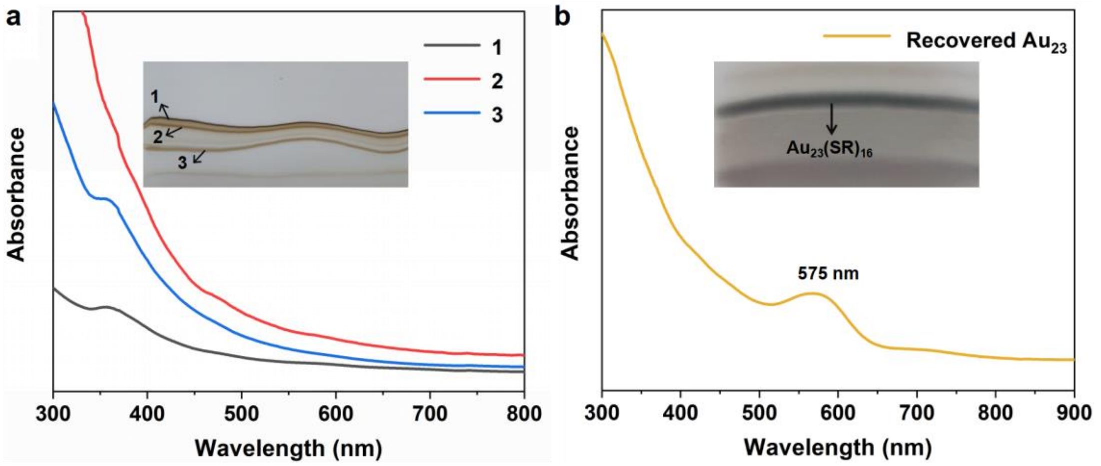

Supplementary Figure 1. (a) UV-vis spectra of the products in the reaction of Au_{23} with butane-2,3-diybis(diphenylphosphine). Inset: PTLC photographs of the reaction of Au_{23} with butane-2,3-diybis(diphenylphosphine). (b) UV-vis spectra of the the product in the reaction of Au_{23} with BINAP. Inset: PTLC photographs of the reaction of Au_{23} with BINAP.

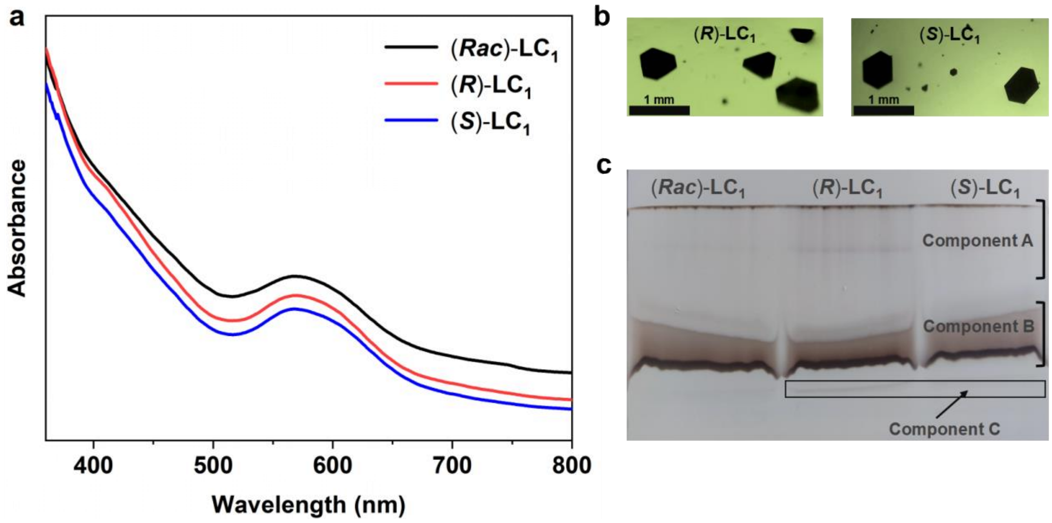

Supplementary Figure 2. (a) UV-vis spectra of (***Rac***)-$\mathbf{LC_1}$, (***R***)-$\mathbf{LC_1}$ and (***S***)-$\mathbf{LC_1}$. (b) Crystal photographs of (***R***)-$\mathbf{LC_1}$ and (***S***)-$\mathbf{LC_1}$. (c) PTLC photographs of the reaction between Au_{23} and $\mathbf{L_1}$.

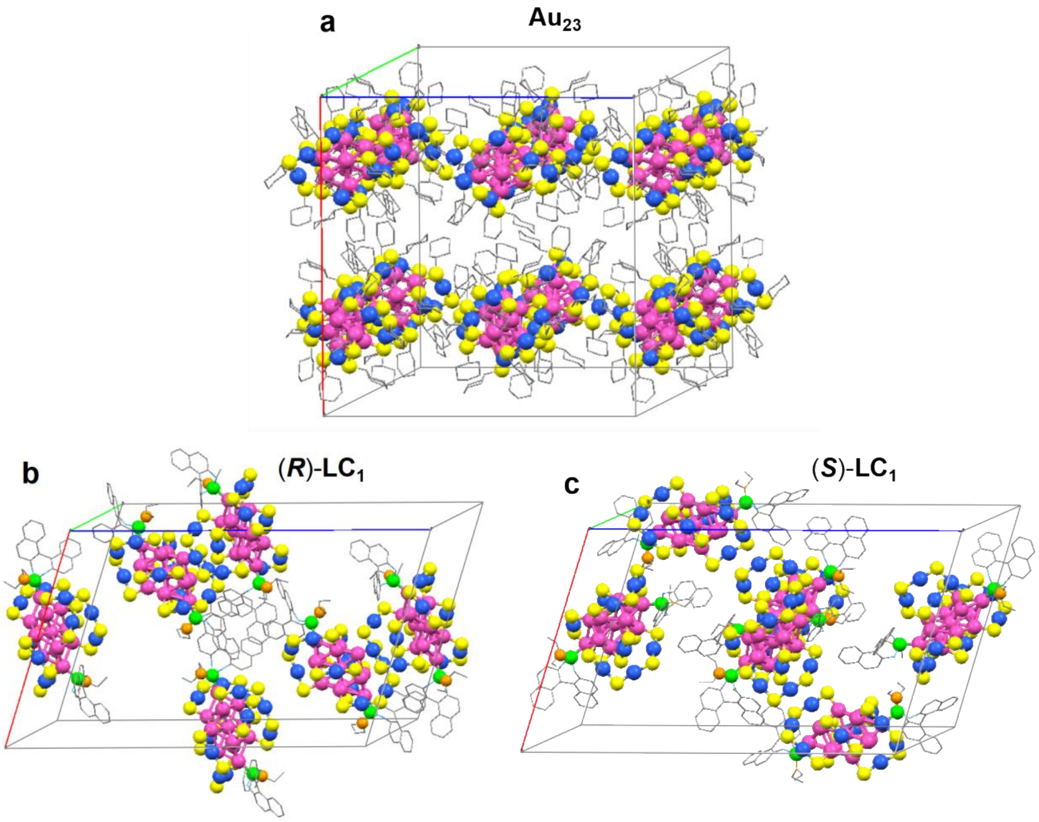

Supplementary Figure 3. Unit cells of Au_{23} (a), (***R***)-$\mathbf{LC_1}$ (b) and (***S***)-$\mathbf{LC_2}$ (c) crystal structures. Color label: Au = pink, blue; S = yellow; P = green; N = orange; O = light blue; C = gray. H atoms are all omited for clearity.

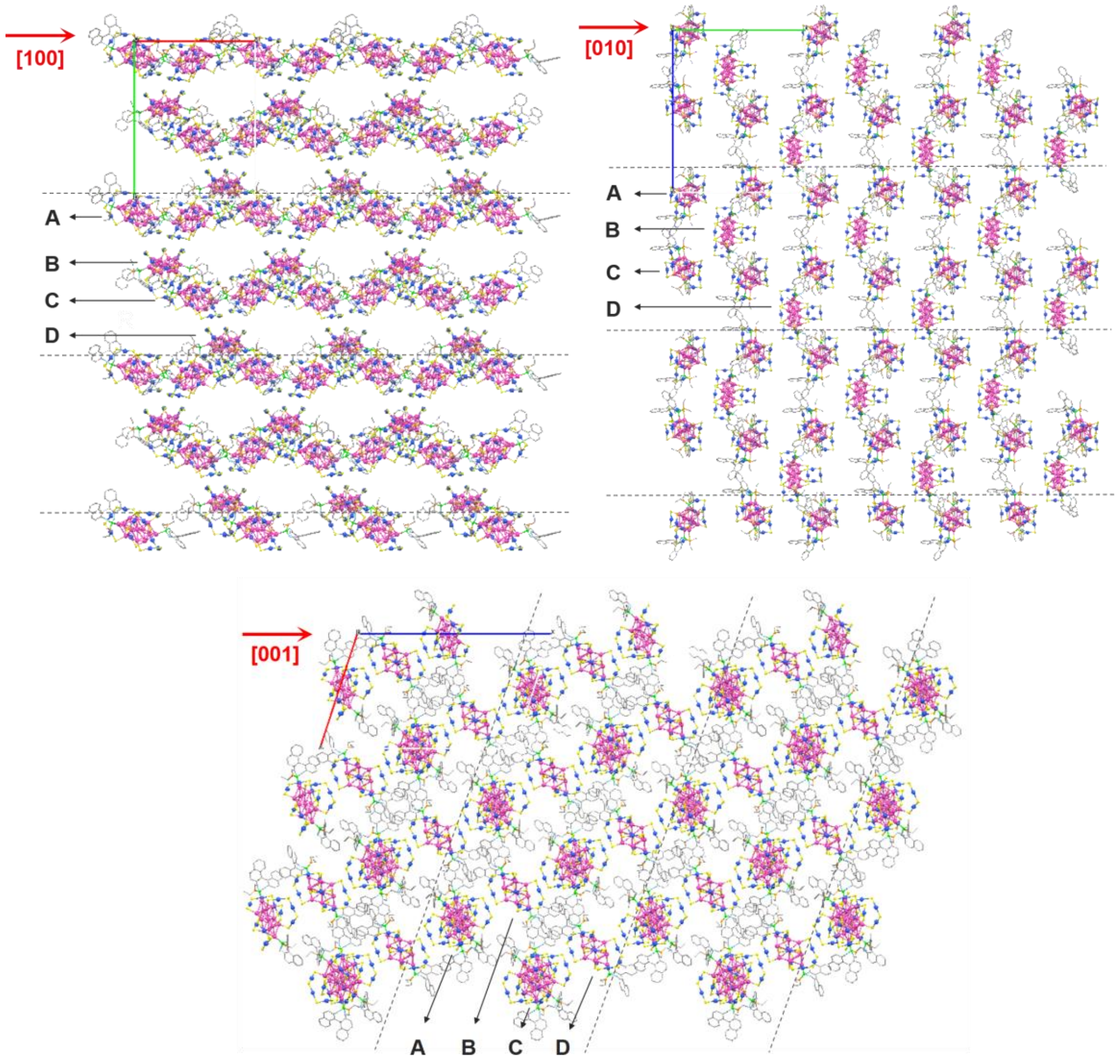

Supplementary Figure 4. Packing mode of (***R***)-**LC$_1$** molecules along the [100], [010] and [001] directions. Color label: Au = pink, blue; S = yellow; P = green; N = orange; O = light blue; C = gray. H atoms are all omited for clearity.

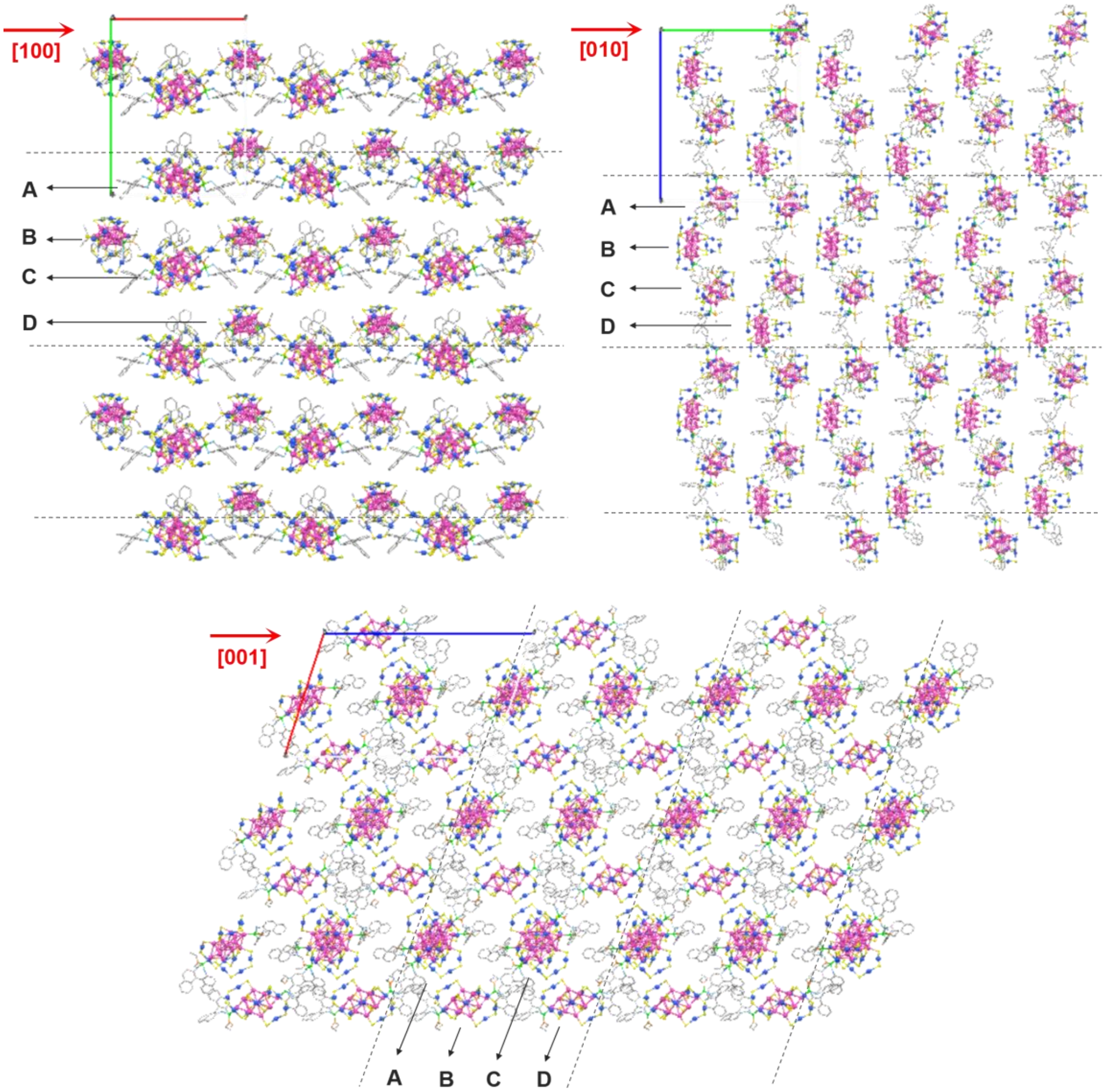

Supplementary Figure 5. Packing mode of (***S***)-**LC**$_\mathbf{1}$ molecules along the [100], [010] and [001] directions. Color label: Au = pink, blue; S = yellow; P = green; N = orange; O = light blue; C = gray. H atoms are all omited for clearity.

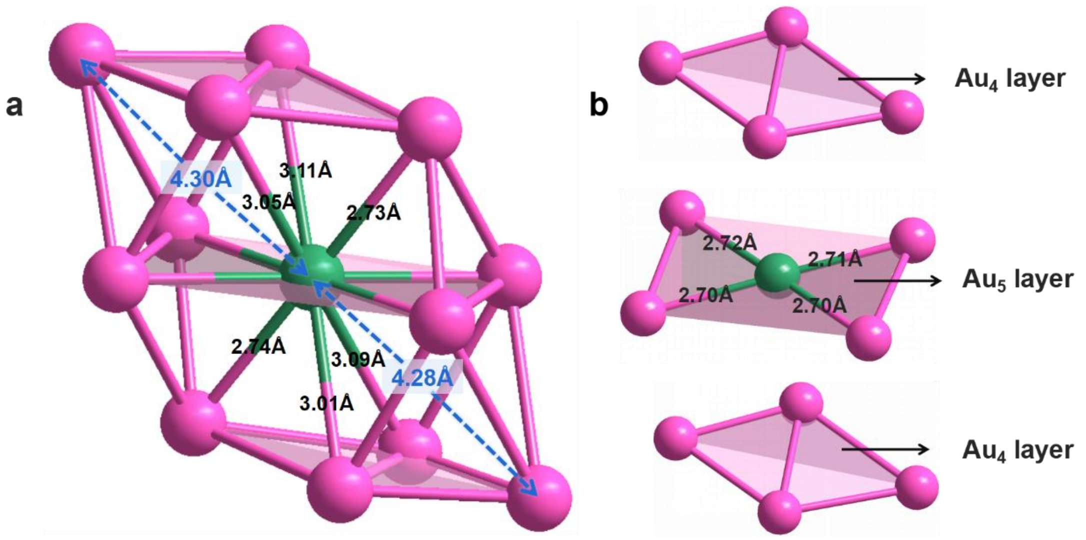

Supplementary Figure 6. (a) Au_{13} kernel of (***R***)-**LC$_1$** and Au–Au distances from the central gold atom to the surrounded gold atoms. (b) Top view of the Au_{13} kernel. Color label: Au = pink, green.

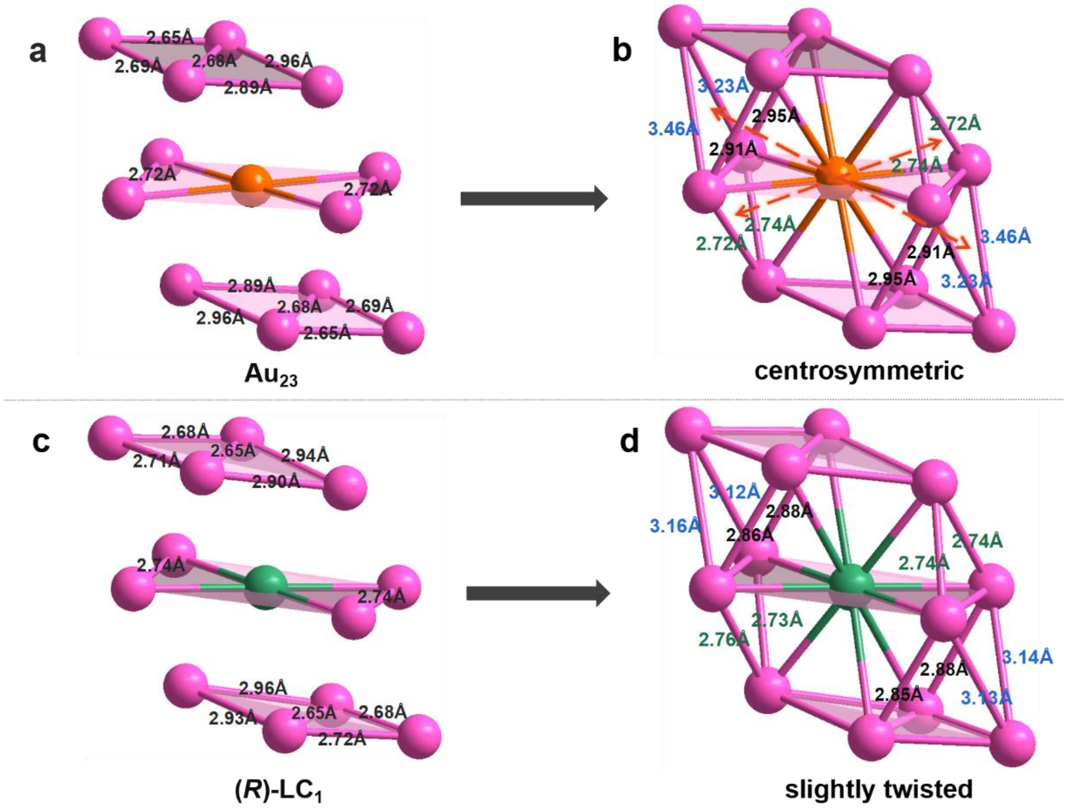

Supplementary Figure 7. Au–Au distances in the Au_{13} kernels of Au_{23} (a and b) and (***R***)-**LC$_1$** (c and d). Color label: Au = pink, orange, green.

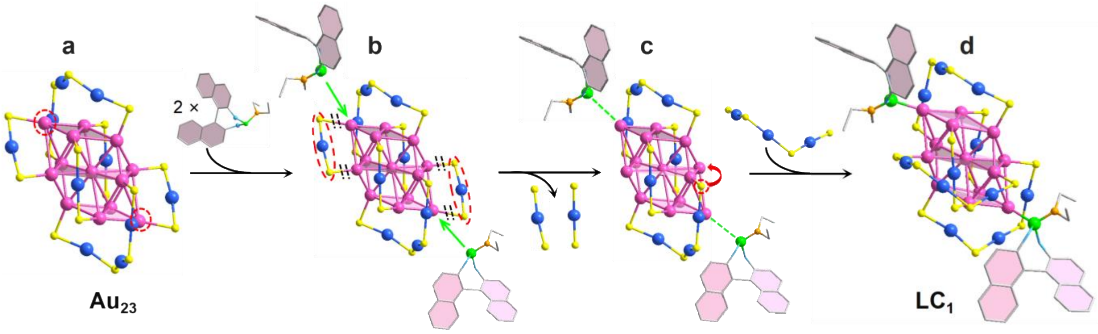

Supplementary Figure 8. The proposed transformation mechanism from Au_{23} to **LC_1**. (a) Structure of Au_{23} (the two red cycle represent the open sites being attacked by **L_1**). (b) **L_1** attack and S-Au-S motifs dissociation. (c) Rearrangement of a -S-Au-S-Au-S-Au-S- motif. (d) Association of a -S-Au-S-Au-S-Au-S- motif.

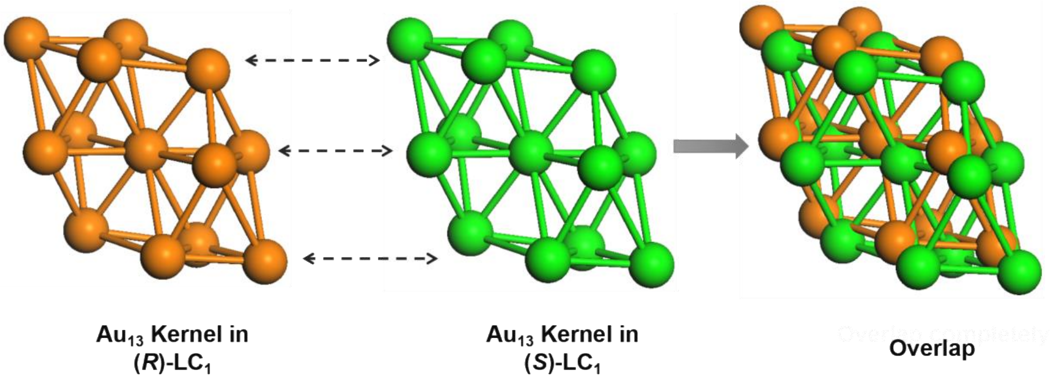

Supplementary Figure 9. The representation of the Au_{13} kernels of (***R***)-**LC_1** and (***S***)-**LC_1**.

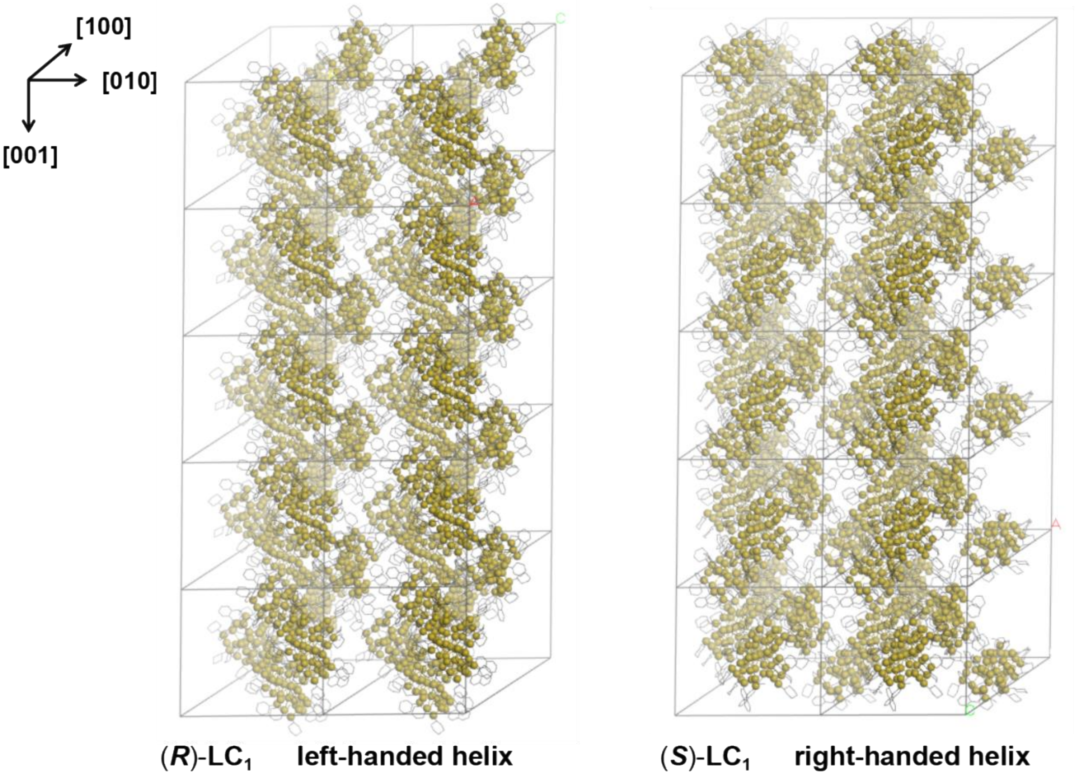

Supplementary Figure 10. The representation of the left- and right-handed helix of (***R***)-**LC**$_1$ and (***S***)-**LC**$_1$.

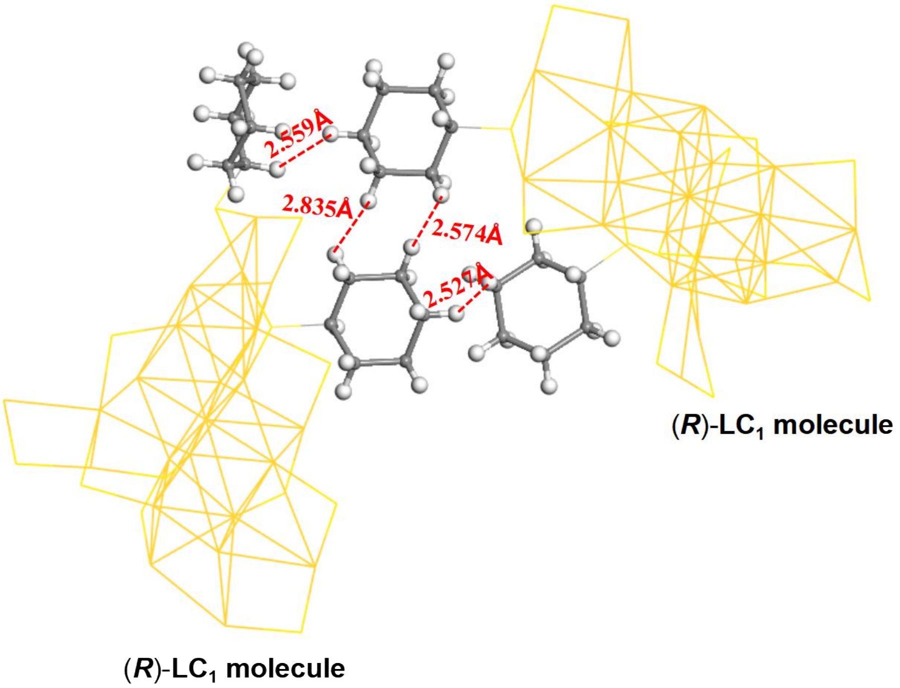

Supplementary Figure 11. The representative H···H interactions between two (***R***)-**LC**$_1$ molecules. Color label: Au, S, P, N, O = yellow; C = gray; H = white.

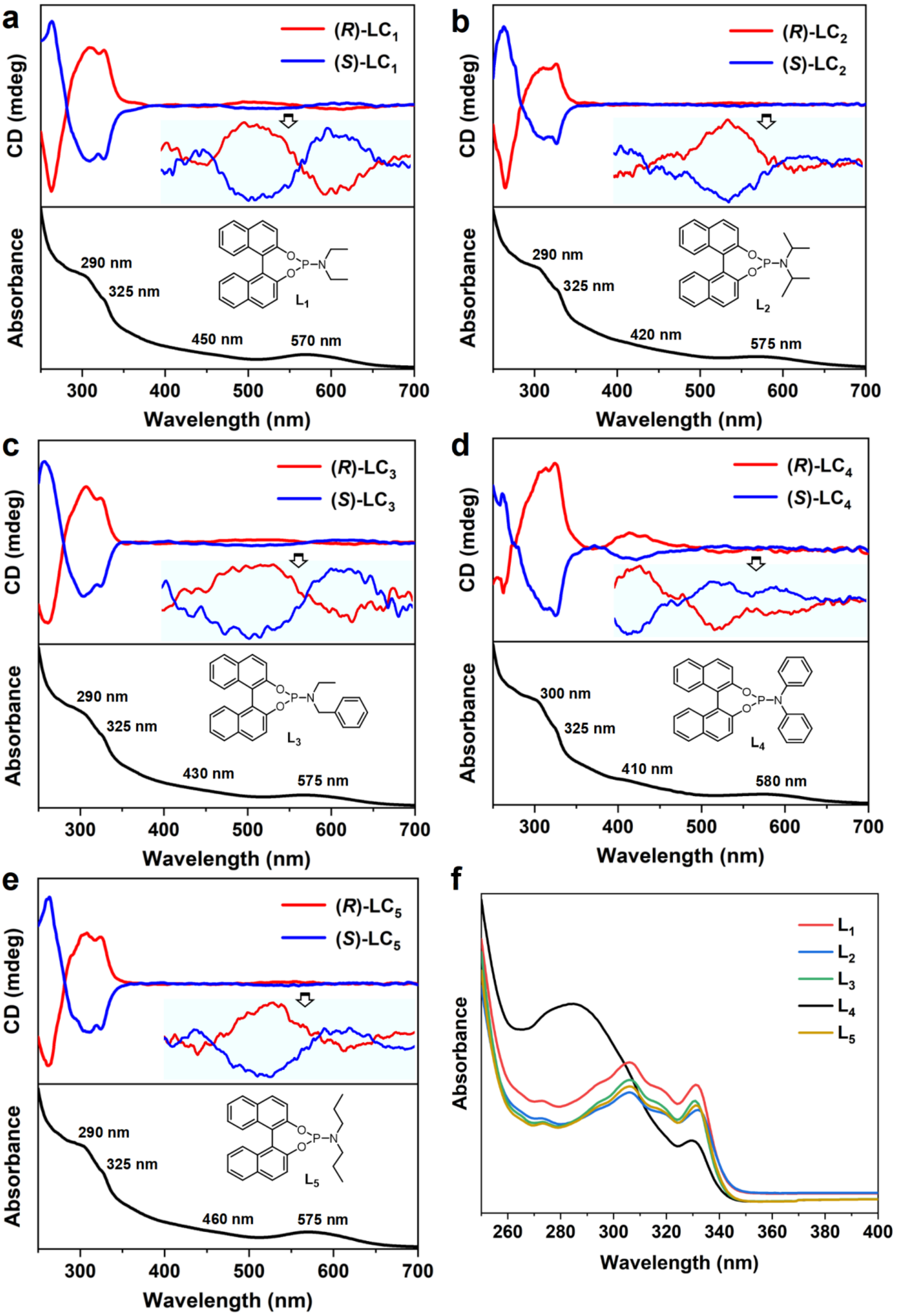

Supplementary Figure 12. (a-e) Combined CD-absorption spectra of **LC_1–LC_5**. (f) UV-vis spectra of **L_1-L_5**. Source data are provided as a Source Data file.

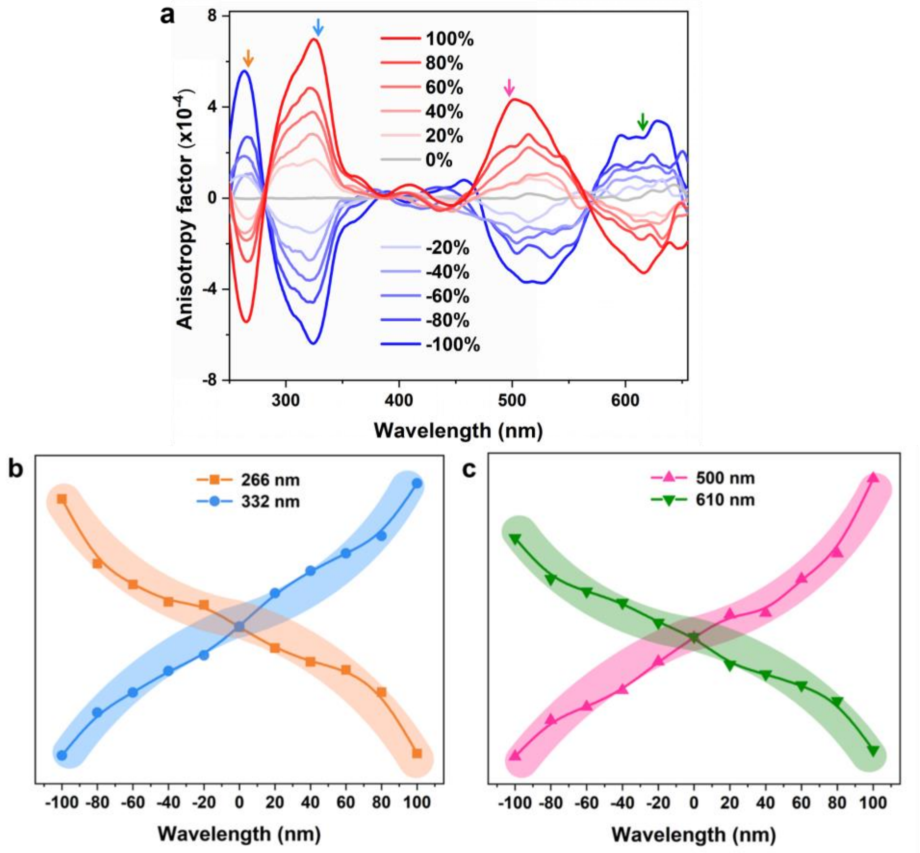

Supplementary Figure 13. (a) Anisotropy factors of **LC_1** prepared by different ee values of **L_1**. (b and c) Representation of the negative nonlinear CD-ee dependence at 266, 322, 500 and 610 nm. The solid and border highlighted colored lines in (b) and (c) are not fitted. They are mere guides to the eye.

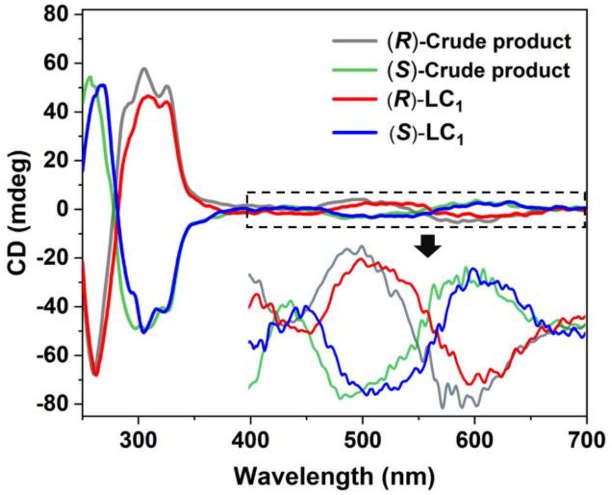

Supplementary Figure 14. CD spectra of the isolated (***R***)- and (***S***)-**LC_1**, and the crude product obtained from the reaction of Au_{23} with (***R***)- and (***S***)-**L_1** (Inset: the enlarged view from 400 to 700 nm, and the magnification factors is 7). Source data are provided as a Source Data file.

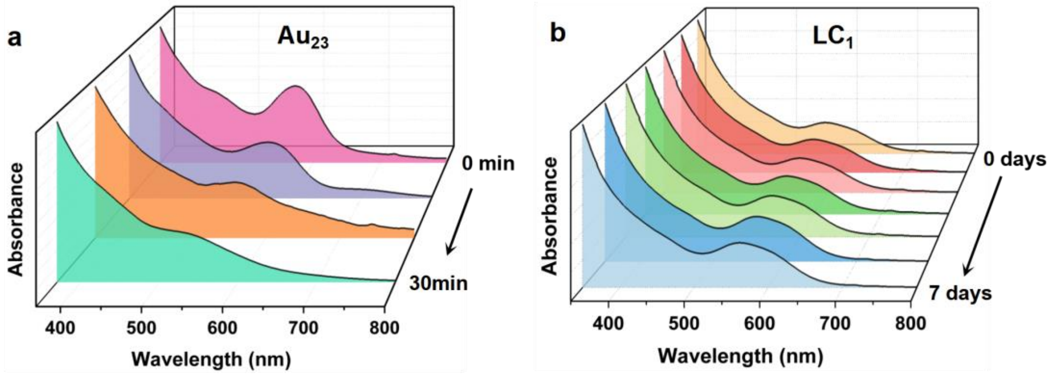

Supplementary Figure 15. Time-dependent UV-vis spectra of Au_{23} and $\mathbf{LC_1}$ at 80℃ (in toluene). Source data are provided as a Source Data file.

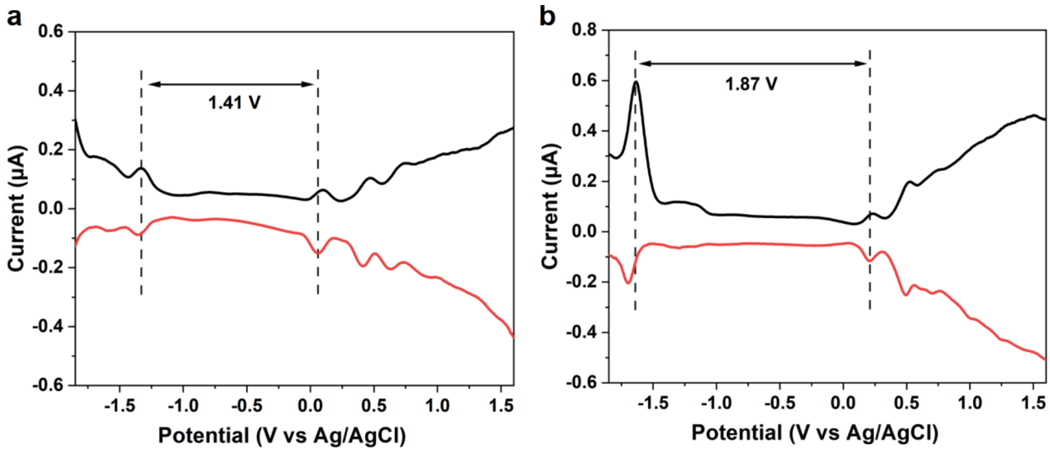

Supplementary Figure 16. DPV spectrum of Au_{23} (a) and $\mathbf{LC_1}$ (b). Source data are provided as a Source Data file.

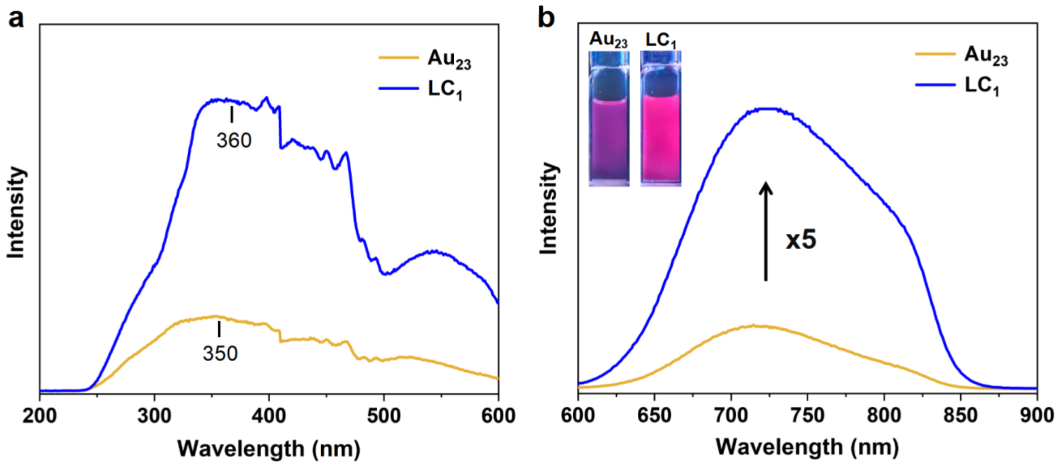

Supplementary Figure 17. (a) Excitation spectra of Au_{23} and $\mathbf{LC_1}$. (b) Emission spectra of Au_{23} and $\mathbf{LC_1}$. The inset shows the photographs of Au_{23} and $\mathbf{LC_1}$ solution under UV lamp (365nm). Source data are provided as a Source Data file.

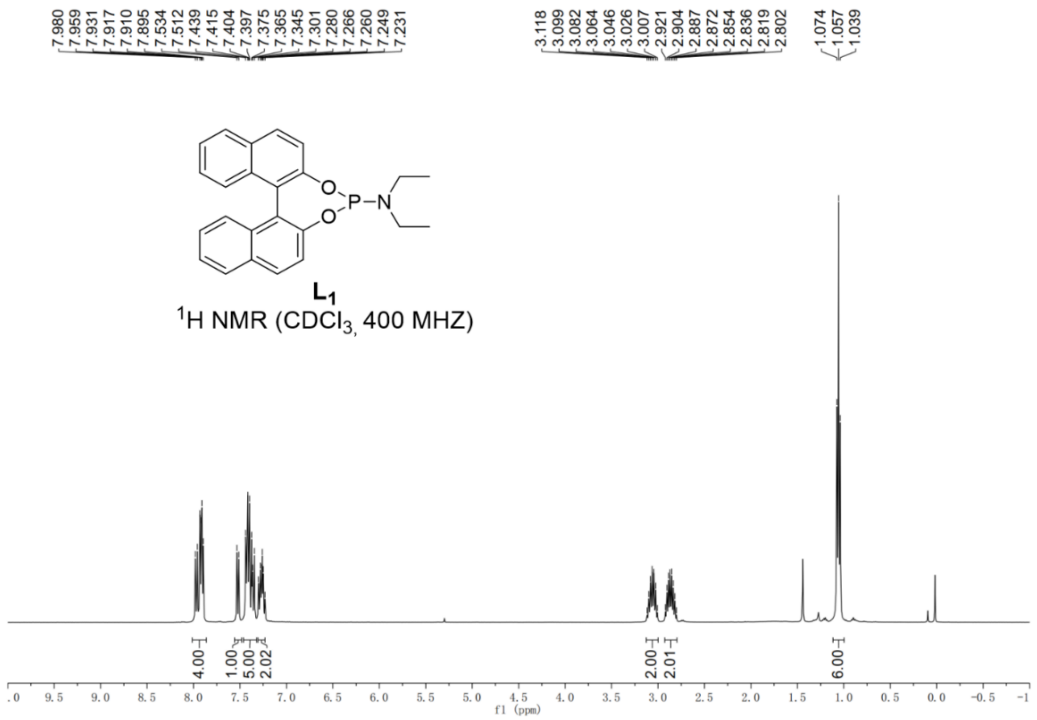

Supplementary Figure 18. ^{1}H NMR spectrum of $\mathbf{L_1}$.

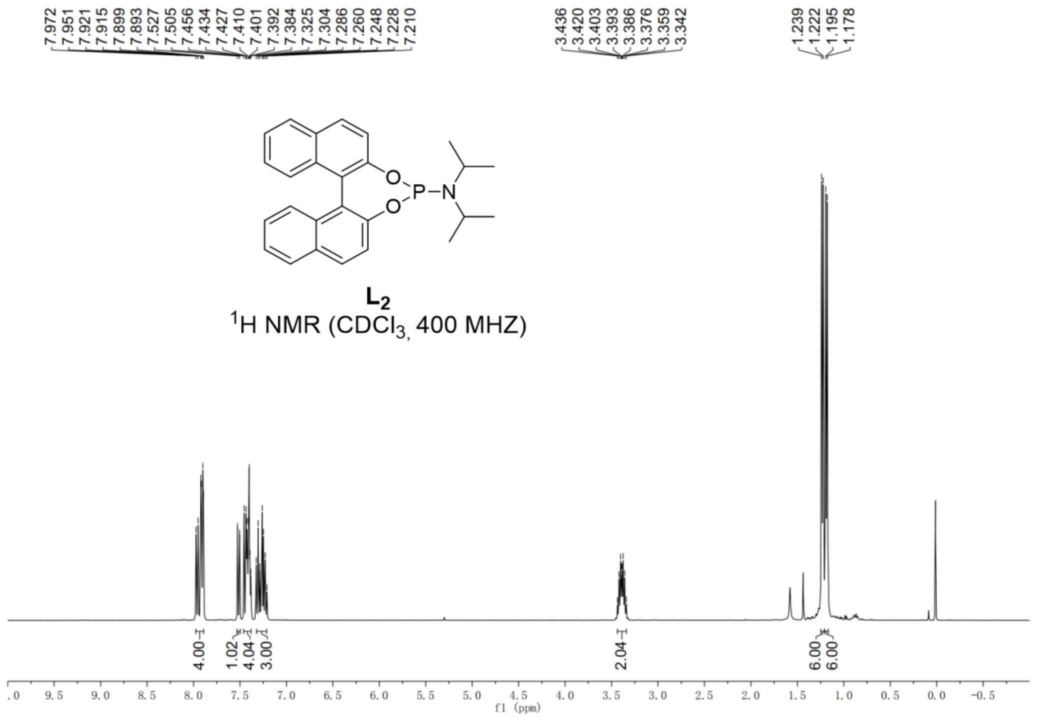

Supplementary Figure 19. ^{1}H NMR spectrum of $\mathbf{L_2}$.

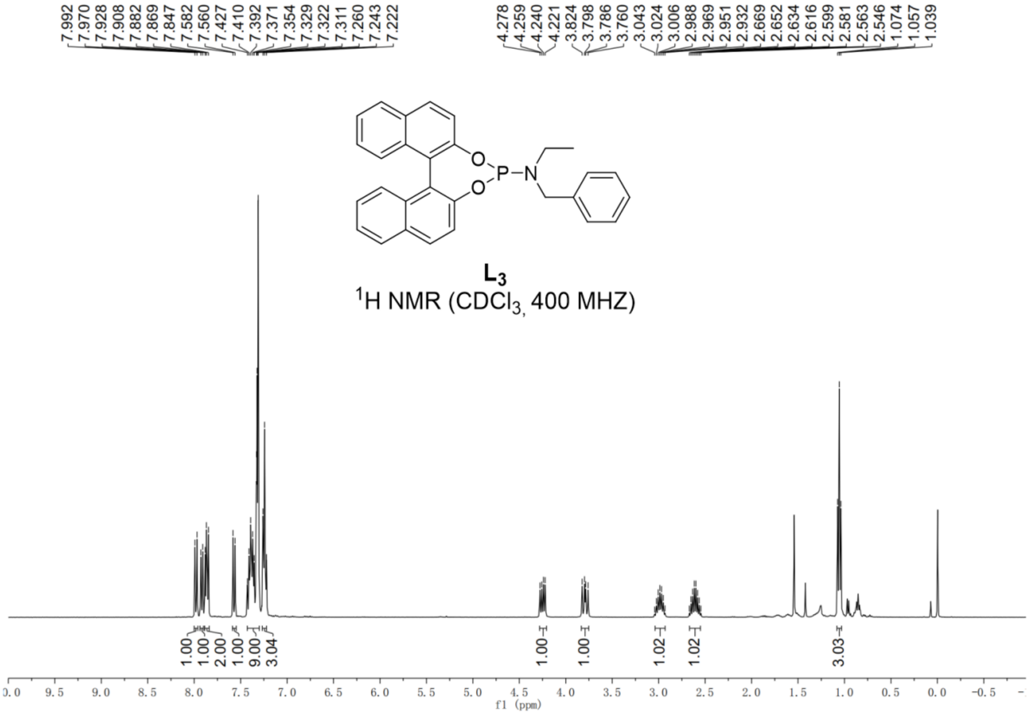

Supplementary Figure 20. ^{1}H NMR spectrum of **L$_3$**.

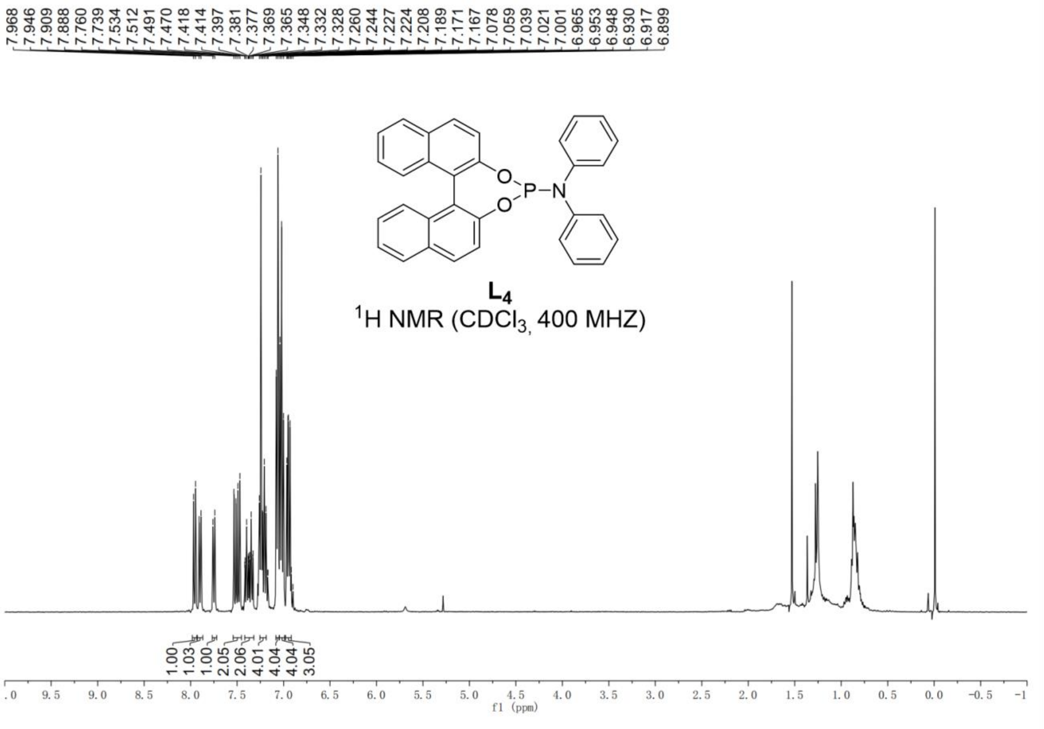

Supplementary Figure 21. ^{1}H NMR spectrum of **L$_4$**.

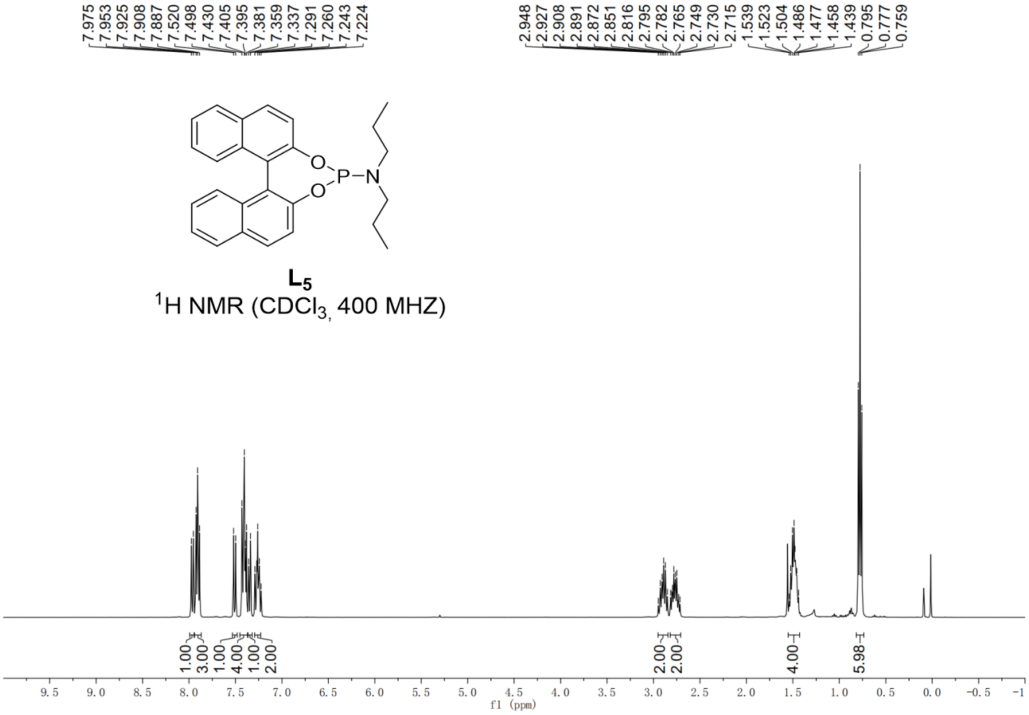

Supplementary Figure 22. ^{1}H NMR spectrum of **L_5**.

Supplementary references

1. Zeng, C., Li, T., Das, A., Rosi, N. L. & Jin, R. Nonsuperatomic $[Au_{23}(SC_6H_{11})_{16}]^-$ nanocluster featuring bipyramidal Au_{15} kernel and trimeric $Au_3(SR)_4$ motif. *J. Am. Chem. Soc.* **135**, 18264-18267 (2013).
2. Ardkhean, R., Roth, P. M. C., Maksymowicz, R. M., Curran, A., Peng, Q., Paton, R. S. & Fletcher, S. P. Enantioselective conjugate addition catalyzed by a copper phosphoramidite complex: computational and experimental exploration of asymmetric induction. *ACS Catal.* **7**, 6729-6737 (2017).
3. Trost, B. M., Silverman, S. M. & Stambuli, J. P. Development of an asymmetric trimethylenemethane cycloaddition reaction: application in the enantioselective synthesis of highly substituted carbocycles. *J. Am. Chem. Soc.* **133**, 19483-19497 (2011).
4. Gao, G.-R., Du, S.-Z., Yang, Y., Lei, X., Huang, H.-Z. & Chang, M.-X. Direct asymmetric reductive amination for the synthesis of (*S*)-rivastigmine. *Molecules* **23**, 2207 (2018).
5. Albat, D., Köcher, A., Witt, J. & Schmalz, H.-G. On the asymmetric iridium-catalyzed n-allylation of amino acid esters: improved selectivities through structural variation of the chiral phosphoramidite ligand. *Eur. J. Org. Chem.* e202200188 (2022).
6. Bernsmann, H., vandenBerg, M., Hoen, R., Minnaard, A. J., Mehler, G., Reetz, M. T., DeVries, J. G. & Feringa, B. L. PipPhos and morfPhos: privileged monodentate phosphoramidite ligands for rhodium-catalyzed asymmetric hydrogenation. *J. Org. Chem.* **70**, 943-951 (2005).
